# *Salix alba* L. Leaf Volatiles Attract *Nematus hequensis* Xiao Adults: Sex-Specific Responses and Optimized Field Trapping Using (*E*)-2-Hexenal and O-Xylene

**DOI:** 10.3390/insects17070714

**Published:** 2026-07-10

**Authors:** Zhenhao Song, Yiqu Chen, Zhaoxu Sun, Qin Li, Xiaoqin Tang, Xueping Lei, Yuan Zhao, Dawei Hong, Jiangcheng Zang

**Affiliations:** 1 Plant Sciences College, Xizang Agricultural and Animal Husbandry University, Nyingchi 860000, China; 202200100052@stu.xza.edu.cn (Z.S.); zkxycyq@xza.edu.cn (Y.C.); 202300100064@stu.xza.edu.cn (Z.S.); 18185487231@163.com (Q.L.); hebeihongdawei@126.com (D.H.); zangjc2008@163.com (J.Z.); 2 Lab of Resource and Applied Insects in the Xizang Plateau, Xizang Agricultural and Animal Husbandry University, Nyingchi 860000, China; 3 Institute of Agricultural Sciences (IAR), Xizang Academy of Agriculture and Animal Husbandry Sciences (XZAAAS), Lhasa 850000, China; 18089082519@163.com (X.L.); xznkyzy08@163.com (Y.Z.)

**Keywords:** *Nematus hequensis* Xiao, *Salix alba* L., host plant volatiles, sex-specific attraction, field trapping optimization

## Abstract

Plant-derived attractants for sawfly pests are rarely available. This study evaluated field trapping of *Nematus hequensis* Xiao adults using 15 volatile compounds from *Salix alba* L. leaves, testing four concentrations, three trap types, two lure cores, two hanging heights, and two trap spacings in a randomized block design (three replicates) during the adult emergence peak in Lhasa, Tibet (2024). Optimal combinations were sex-specific: for females, large boat-shaped traps suspended at 1 m with slow-release bottle lures containing 10 μg/μL *o*-Xylene captured 62 individuals; for males, the same configuration but with 10 μg/μL (*E*)-2-Hexenal captured 265 individuals. Trap spacing (10 m vs. 15 m) did not significantly affect daily capture. These results provide a practical, environmentally friendly monitoring tool for *N. hequensis*. To our knowledge, within the genus *Nematus*, this study is the first to develop a plant-volatile attractant based on host leaf volatiles.

## Simple Summary

Willow trees are important for riverbank protection, wood production, and landscaping, but they are increasingly damaged by leaf-eating insects. One such pest, the sawfly *Nematus hequensis* Xiao, has caused severe defoliation in Tibet and other regions of China. Current control relies heavily on chemical insecticides, which can harm the environment. In this study, we tested 15 natural chemicals released by willow leaves to see if they could attract adult sawflies to traps. Field experiments in Lhasa, Tibet, showed that males and females responded to different chemicals: males were most attracted to (*E*)-2-hexenal, while females preferred o-xylene. We also found that the best trap design was a large boat-shaped trap hung at 1 m above ground, with a slow-release lure at a specific concentration. Trap spacing did not affect capture rates. These findings provide a practical, environmentally friendly tool for early detection of sawfly outbreaks, which could help reduce chemical spraying and protect willow trees.

## 1. Introduction

*Salix alba* L. (white willow) is widely planted worldwide for riparian restoration, biomass production, and ornamental use, with a natural range spanning Europe, Asia, and North Africa, and introductions on all continents except Antarctica. However, its expansion has facilitated the spread of specialist herbivores. Among them, sawflies (Hymenoptera: Tenthredinidae) are particularly damaging due to gregarious defoliation during their larval stages, which can completely strip host trees and reduce growth, timber quality, and ecosystem services. One emerging pest is *Nematus hequensis* Xiao, first described from the Hequ region of China [1]. Over the past three decades, it has gradually expanded its range and now causes increasing damage in several Chinese provinces, including Liaoning, Inner Mongolia, Beijing, and most recently the Tibet Autonomous Region [2]. Although currently reported only in China, the global distribution of *S. alix* hosts—including *S. alba*, *Salix matsudana*, and *hybrid poplars*—raises concerns about its potential as an invasive pest, especially under climate change scenarios that may expand suitable habitat at higher latitudes and altitudes [3]. This highlights the urgent need for effective, low-environmental-impact monitoring and control tools that can be deployed preemptively.

Belonging to the order Hymenoptera and family *Tenthredinidae*, *N. hequensis* is a phytophagous pest that feeds on the leaves of *S. matsudana* Koidz, *S. alba* Tristis, *S. alba* L., *Populus* × *beijingensis* W. Y. Hsu, and other plants [4]. The insect was discovered and named in 1990 [1]. Currently, *N. hequensis* is extensively found in Liaoning (Zhanggutai), Inner Mongolia (Hulunbeier), Beijing, and Xizang (Lhasa), among other places in China [5]. The insect damages host plants by feeding on their leaves during the larval stage, and it exhibits characteristics that include rapid reproduction, high population density, significant food consumption, and causes substantial damage [2]. Current control relies almost exclusively on chemical insecticides, mainly organophosphates and pyrethroids [6]. Although no resistance has been reported to date, long-term pesticide use inevitably leads to environmental pollution, non-target effects (e.g., on pollinators and natural enemies), resistance development, and health risks to applicators and nearby residents [7,8]. There is therefore a pressing need for alternative, sustainable management methods.

Plant-based attractants offer a greener alternative. The use of host plant volatiles to lure pests into traps-known as attract-and-kill or monitoring-has been successfully developed for many insect groups, including weevils, moths, and fruit flies [9]. The efficacy of such lures depends on multiple factors: volatile composition and concentration, lure type (release rate, longevity), trap design (color, shape, entrance size), trap density and placement height, as well as environmental conditions (temperature, wind, humidity). For sawflies, however, very few plant-volatile attractants have been developed, and none exist for *N. hequensis*. Previous studies in our laboratory identified 15 volatile compounds from *S. alba* leaves with relative contents ≥ 1% and demonstrated that several of these compounds elicited electroantennographic and behavioral responses in *N. hequensis* adults under laboratory conditions [10]. However, field validation and optimization of trapping parameters have not been conducted.

The specific objectives of this study were: (1) to screen the 15 *S. alba* leaf volatiles at four concentrations for field attractiveness to *N. hequensis* adults; (2) to determine the effects of trap type (large boat-shaped vs. triangular), hanging height (1.0 vs. 1.5 m), and trap spacing (10 vs. 15 m) on capture efficiency; (3) to identify the optimal combination for each sex; and (4) to provide a practical, environmentally friendly monitoring tool for early warning, and to inform future evaluation of mass trapping feasibility for this emerging sawfly pest.

## 2. Materials and Methods

### 2.1. Field-Trapping Experiment Locations and Times

The field-trapping experiment was set up at the Agricultural Research Institute of the Lhasa City Agricultural and Forestry Science Academy, Tibet Autonomous Region (China; 29°38′26″ N, 91°2′5″ E), at an elevation of 3650 m above sea level. The site covers an area of approximately 4 ha. To ensure accuracy, no insecticides or control measures were used in this area before or during the experiment. The experiment was conducted from 25 August to 28 September 2024, during the peak outbreak period of adult *N. hequensis* (the adult emergence period may vary in different years, regions, or areas due to the region, altitude, temperature, humidity, and light). During the experimental period (25 August–28 September 2024), the mean temperature was 14.2 °C (range 8.5–21.0 °C), mean relative humidity 63%, and total rainfall 48 mm (data from Lhasa Meteorological Bureau, weather.cma.cn).

### 2.2. Preparation of Attractant Lures

This study builds on our previous research [10], in which the 15 volatile compounds listed in Table 1 were identified and quantified from *S. alba* leaves using GC-MS. The present study focuses on field validation and optimization of these compounds as attractants for *N. hequensis* adults, which was not conducted in our earlier work. Fifteen volatile compounds (purity ≥ 97%, suppliers listed in Table 1) were each diluted in *n*-hexane (≥99.5%; Tianjin Kermel Chemical Reagent Co., Ltd., Tianjin, China) to four concentrations: 100, 10, 1, and 0.1 μg/μL. For each concentration, 10 μL of the diluted volatile was mixed with 10 μL of natural honey (from *Lepidotrigona arciferal*, provided by the Agricultural Research Institute, Lhasa, China) as a slow-release carrier. The mixture (20 μL total) was injected into a polyethylene bottle lure (slow-release bottle core, model YL-16mL, Zhongjie Sifang Co., Beijing, China) using a microsyringe. Blank control lures received 20 μL of *n*-hexane + honey mixture without any volatile compound. All lures were individually wrapped in aluminum foil, sealed with Parafilm, and stored at −20 °C for a maximum of 7 days. Lures were brought to room temperature 30 min before field deployment. Preliminary laboratory measurement at 25 °C indicated an average release rate of 0.42–0.68 μg/day for the tested compounds at 10 μg/μL.

### 2.3. Information on Blank Attractant Cores and Traps

As shown in Table 2, the three trap types and three lure core types listed above were tested in preliminary field trials (one week, three replicates per treatment) conducted in early August 2024. Based on these preliminary results, bucket traps and eraser/bag lures captured negligible numbers of adults (<30 total across all treatments) and were therefore excluded from the main randomized block experiment. Only large boat-shaped traps, triangular traps, and slow-release bottle lures were carried forward into the main experimental design described below. Three types of traps were used: large boat-shaped, triangular, and bucket-shaped traps (Zhongjie Sifang Co., Ltd., Beijing, China; model numbers YL-LWT-1, YL-DT-2, and BT-L-P2.0, respectively). The attractant cores included eraser-type slow-release, slow-release bag, and slow-release bottle cores (Zhongjie Sifang Co., Ltd.; model numbers Y, YL, and YL-2ml, respectively).

### 2.4. Field Trapping Experimental Design

The experiment followed a randomized complete block design with three blocks (replicates), each block separated by at least 200 m to avoid interference. Within each block, traps were arranged in a grid with a spacing of either 10 m or 15 m (two density treatments). A 5 m buffer zone of untreated *S. alba* trees surrounded each block.

For each volatile compound, concentration, trap type, lure core type, and hanging height (1.0 m or 1.5 m) were tested. Blank control traps (containing no volatile) were included in all configurations to account for physical attraction.

Traps were rotated weekly within each block to minimize position effects. Sticky boards were replaced weekly, and water in bucket traps replenished. The experiment lasted four consecutive weeks (25 August–28 September 2024), with counts recorded daily during the first three days of each week, then averaged per week. Each treatment combination was therefore replicated three times (once per block) and over three weekly measurements.

The field experiments were conducted as a series of three sequential sub-experiments rather than as a single fully factorial design:

(1) Compound and concentration screening: All 15 volatile compounds at four concentrations (100, 10, 1, and 0.1 μg/μL) were tested using large boat-shaped traps suspended at 1.0 m with slow release bottle lures. This experiment identified the most attractive compounds and optimal concentrations for each sex.
(2) Trap type comparison: Using the best performing compounds ((*E*)-2-hexenal for males, o-xylene for females) at the optimal concentration (10 μg/μL), we compared large boat-shaped traps versus triangular traps at 1.0 m height.
(3) Hanging height and trap spacing comparison: Using the optimal trap type (large boat-shaped) and lure configuration (10 μg/μL), we compared hanging heights (1.0 vs. 1.5 m) and trap spacings (10 vs. 15 m).

In each sub-experiment, the factors not being tested were held constant to isolate the effect of the target variable. Within each sub-experiment, the design was randomized complete block with three blocks (spatial replicates).

### 2.5. Field Population Monitoring of N. Hequensis Adults

From July 22 to September 23 in 2023 and 2024, adult *N. hequensis* abundance was monitored in the same experimental site. Six plots (each 0.5 ha) were established. In each plot, five *S. alba* trees (10–15 years old, 5–8 m height) were randomly selected. At each tree, a sweep net (40 cm diameter) was used to capture adults within a 5 m radius around the trunk for 5 min. Captured adults were counted and sexed. Sampling was conducted weekly in the morning (China, 10:00–12:00) under sunny conditions, with five repetitions (five separate sampling rounds) per monitoring year.

### 2.6. Statistical Analysis

Data are presented as mean ± standard error (SE). All statistical analyses were performed using SPSS v.20.0 (IBM, Armonk, NY, USA) and R v.4.2.1 (R Core Team, 2022, Vienna, Austria) with the ‘lme4’ and ‘emmeans’ packages.

Because the response variable was insect count data, which often exhibit overdispersion, we fitted generalized linear mixed models (GLMMs) with a negative binomial distribution and a log-link function. In all models, block was treated as a random effect to account for spatial variability among the three replicate blocks, while treatment factors (volatile compound, concentration, trap type, lure type, hanging height, and trap spacing) were fitted as fixed effects. Weekly means per trap were aggregated across the four-week experimental period and analyzed as a single seasonal mean per trap, with block as the random effect to account for spatial variability. This approach was justified because trap rotation and weekly board replacement minimized positional bias, and preliminary analysis showed no significant week-to-week variation in treatment ranking. When significant fixed effects were detected, post hoc pairwise comparisons were performed on AIC values. All figures and significance markers (letters and asterisks) were generated based on the GLMM/Tukey HSD results. Model assumptions (normality of residuals and homogeneity of variance) were checked using diagnostic Q-Q plots and residual-vs-fitted plots. The negative binomial distribution was selected over Poisson based on Akaike Information Criterion (AIC) values, confirming that the data exhibited overdispersion relative to a Poisson distribution and were therefore better modeled by a negative binomial GLMM.

Rmed using Tukey’s Honestly Significant Difference (HSD) test (α = 0.05) was used to control family-wise error rates. For factors with only two levels (e.g., height: 1.0 vs. 1.5 m; spacing: 10 vs. 15 m), we report the GLMM fixed-effect Wald F-tests directly.

Prior to model fitting, we confirmed that the negative binomial distribution provided a better fit than Poisson distribution-based.

## 3. Results

### 3.1. Effect of Attractant Concentration on Trapping Amount

**Figure 1:**
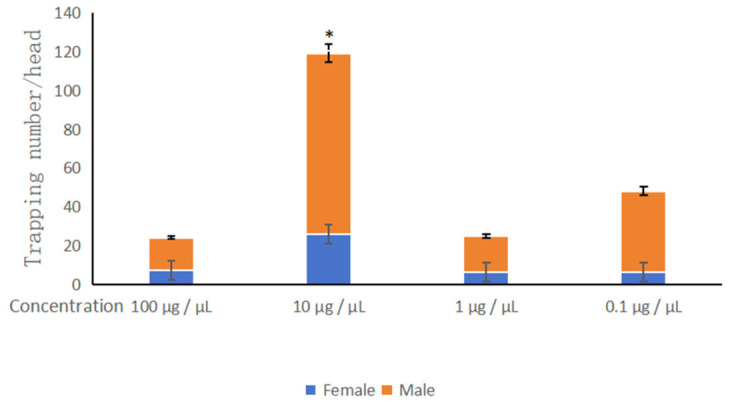
Trapping amounts of the attractant at different concentrations. Note: “*” (mean ± SE) indicates significant differences (α = 0.05) (*n* = 3).

Different concentrations of plant-derived attractants did not cause a linear concentration-dependent increase in trapping efficiency (Figure 1). However, the trapping quantity at a concentration of 10 μg/μL (119 individuals per day on average) was significantly higher than that at other concentrations.

The concentration of attractants significantly affected daily capture (GLMM Wald F_3,8_ = 12.7, *p* < 0.001). At 10 μg/μL, the mean capture was 119 ± 9.3 adults/day (*n* = 3), which was significantly higher than at 100 μg/μL (32 ± 5.1), 1 μg/μL (28 ± 4.6), and 0.1 μg/μL (41 ± 6.2) (Tukey’s HSD test, *p* <0.05). Notably, the highest concentration (100 μg/μL) did not yield the highest capture, indicating a non-linear dose–response relationship (Figure 1).

### 3.2. Effect of Trap Density on Trapping Amount

**Figure 2:**
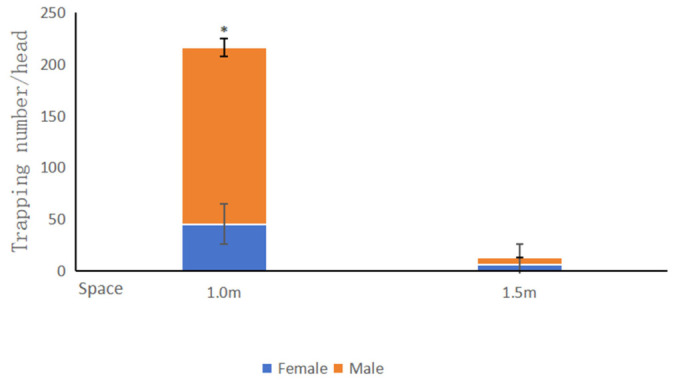
Trapping amounts of the traps at different heights. Note: “*” (mean ± SE) indicates significant differences (α = 0.05) (*n* = 3).

Trap spacing (10 m vs. 15 m) did not significantly affect daily capture for either females (GLMM Wald F_1,4_ = 2.31, *p* = 0.20) or males (GLMM Wald F_1,4_ = 1.89, *p* = 0.24), with average captures of 49 and 33 females per trap, and 175 and 142 males per trap, respectively (Figure 2). Given the lack of statistical difference and lower material cost, a spacing of 10 m (one trap per 100 m^2^) is recommended for practical application.

### 3.3. Effect of Trap Suspension Height on Trapping Amount

Hanging height had a strong effect on capture (GLMM Wald F_1,4_ = 34.5, *p* = 0.004). Traps suspended at 1 m captured 216 ± 18.2 adults/day, whereas those at 1.5 m captured only 14 adults/day (Figure 2). The manufacturer’s recommended height (1.5 m) was therefore ineffective for this species under local conditions.

### 3.4. Effects of Different Lures and Traps on Trapping Amount

#### 3.4.1. Large Boat-Shaped Trap

**Figure 3:**
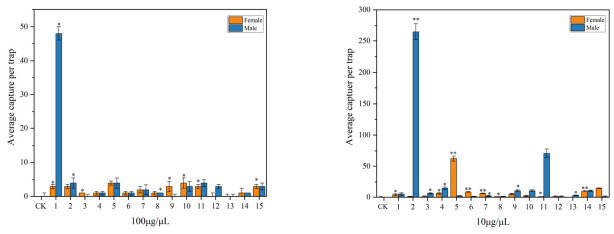

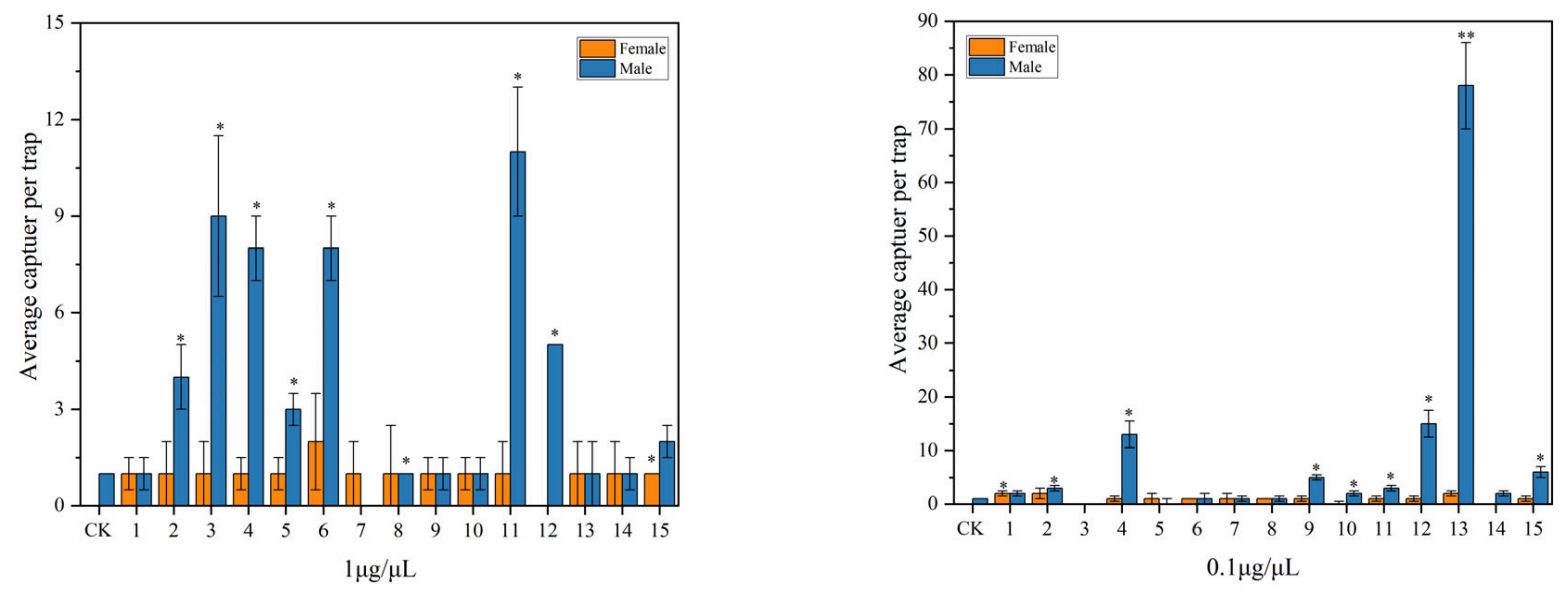
Field-trapping effect of the large boat-shaped trap. 1: dimethyl sulfide; 2: (*E*)-2-Hexenal; 3: ethylbenzene; 4: *p*-Xylene; 5: *o*-Xylene; 6: styrene; 7: benzaldehyde; 8: furan, 2-pentyl-; 9: 3-hexen-1-ol, acetate, (*Z*); 10: benzyl alcohol; 11: 2-hydroxybenzaldehyde; 12: 1,6-dioxaspiro[4.4]nonane, 2-ethyl; 13: butanoic acid, 3-hexenyl ester, (*Z*); 14: cis-3-hexenyl isovalerate; 15: pentadecanal. Note: Asterisks indicate significant differences based on Tukey’s HSD test following GLMM analysis (* *p* < 0.05; ** *p* < 0.01).

As shown in Figure 3, at a concentration of 100 μg/μL, dimethyl sulfide attracted 48 male and 3 female adults. At a concentration of 10 μg/μL, (*E*)-2-Hexenal had the strongest attractive effect on male adults, attracting 265 individuals, while also attracting a single female adult. 2-Hydroxybenzaldehyde attracted 71 male and 1 female adults, and *o*-Xylene attracted 62 female and 2 male adults. At a concentration of 1 μg/μL, most volatiles exhibited weak attractive effects on both female and male adults. At a concentration of 0.1 μg/μL, butanoic acid, (*Z*)-3-hexenyl ester exerted the strongest attractive effect on male adults, attracting 78 individuals, while also attracting two female adults. Overall, at a concentration of 10 μg/μL, the number of attracted female and male adults was the highest, with 107 female and 483 male adults. This indicates that at higher concentrations, the large boat-shaped trap exhibited good trapping effects on both female and male adults.

#### 3.4.2. Triangle Trap

**Figure 4:**
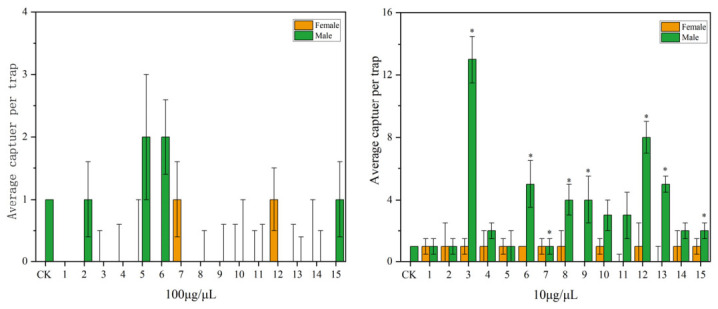

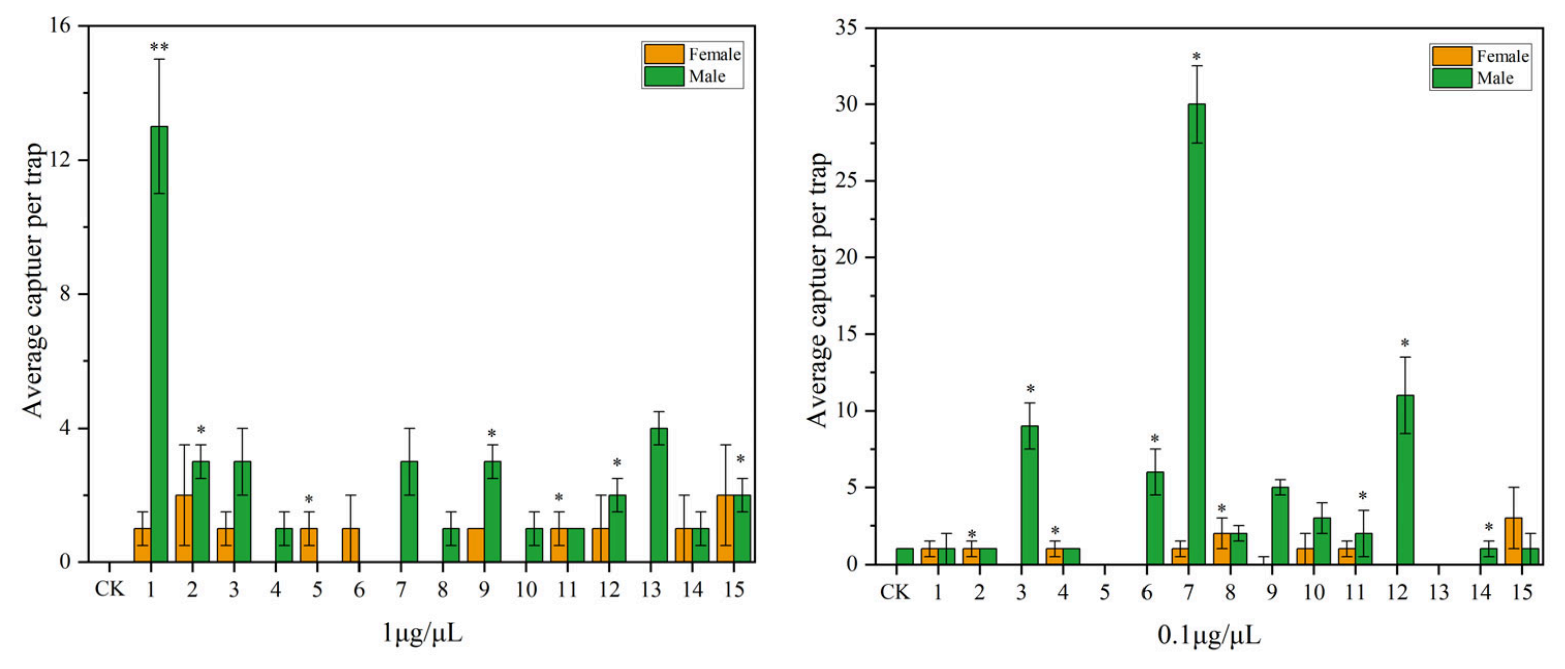
Field-trapping effect of the triangular traps. 1: dimethyl sulfide; 2: (*E*)-2-Hexenal; 3: ethylbenzene; 4: *p*-Xylene; 5: *o*-Xylene; 6: styrene; 7: benzaldehyde; 8: furan, 2-pentyl-; 9: 3-hexen-1-ol, acetate, (*Z*); 10: benzyl alcohol; 11: 2-hydroxybenzaldehyde; 12: 1,6-dioxaspiro[4.4]nonane, 2-ethyl; 13: butanoic acid, 3-hexenyl ester, (*Z*); 14: cis-3-hexenyl isovalerate; 15: pentadecanal. Note: Asterisks indicate significant differences based on Tukey’s HSD test following GLMM analysis (* *p* < 0.05; ** *p* < 0.01).

Compared with the control group, in the experimental group at a concentration of 100 μg/μL, all volatiles exhibited weak trapping effects on both female and male adults (Figure 4). At a concentration of 10 μg/μL, ethylbenzene had the strongest trapping effect on male adults, attracting 13 individuals, with no female adults. At a concentration of 1 μg/μL, all volatiles exerted weak trapping effects on both female and male adults. At a concentration of 0.1 μg/μL, benzaldehyde had the strongest attractive effect on male adults, attracting 30 individuals, while attracting no female adults. Overall, at a concentration of 0.1 μg/μL, a relatively high number of male adults were trapped (67 individuals), whereas the number of female adults was lower. This indicates that the triangular trap had a stronger trapping effect on male adults at this concentration, whereas the effect it had on female adults was relatively weaker.

#### 3.4.3. Bucket Traps and Eraser/Bag Lures

In preliminary field tests conducted prior to the main experiment, bucket traps (model BT-L-P2.0) and eraser-type (model Y) and bag-type (model YL) lure cores captured fewer than 30 adults in total across all volatile compounds and concentrations tested. These configurations were therefore excluded from the main randomized block experiment, and no further quantitative data are presented. This outcome is consistent with the poor performance of these trap and lure types observed in other sawfly trapping studies (see Discussion Section 4.8).

### 3.5. Dynamic Monitoring of the N. Hequensis Adult Population in the Field

**Figure 5:**
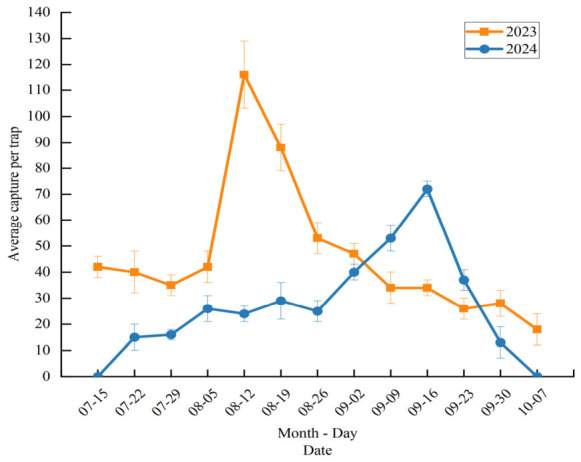
Occurrence dynamics of the *N. hequensis* adult population from 2023 to 2024.

The survey results of the population dynamics of *N. hequensis* adults in the test area (Figure 5) showed that during the monitoring period in 2023, one major peak (12 August) and one smaller peak (19 August) were observed, which occurred on 12th August and 19th August, respectively. This may be related to the high temperatures in the region during this period (Website for Weather Inquiry in Lhasa City: weather.cma.cn). During the monitoring period in 2024, only one small peak period of emergence was observed on 15th September (compared with that in 2023). The survey found that during the *N. hequensis* emergence period, the region experienced continuous rainfall and low-temperature weather, which might have been responsible for the absence of a peak emergence period. Overall, the population density in 2024 was significantly lower than that in 2023, and duration of the emergence period was shorter. Despite the lower overall density in 2024, preliminary observations showed that monthly population fluctuations were similar to those in 2023, suggesting that population size within the same year did not affect the comparison of treatment outcomes.

## 4. Discussion

### 4.1. Non-Linear Concentration–Response and Ecological Implications

The non-linear dose–response (peak attraction at 10 μg/μL, decline at 100 μg/μL) observed in this study aligns with the sensory adaptation and saturation hypothesis documented in many phytophagous insects [9,11]. Although our field data alone do not allow us to identify the exact behavioral mechanism underlying the reduced captures at the highest concentration, a plausible explanation is peripheral olfactory receptor saturation or adaptation. This interpretation is physiologically supported by our previous Electroantennogram (EAG) recordings, which showed that antennal responses to these volatile compounds do not increase linearly but instead follow a threshold-dependent pattern [10]. However, we acknowledge that other factors—such as altered release rates from lures at high concentrations, changes in the spatial structure of the volatile plume, or even repellent effects—could also contribute to the observed decline. Therefore, we present receptor saturation as a possible mechanism, rather than the definitive cause, and emphasize that further behavioral and neurophysiological studies are needed to disentangle these confounding effects.

### 4.2. Sex-Specific Attraction: Chemical Ecology of Host Finding

The strong attraction of *N. hequensis* female adults to *o*-Xylene (12.4% relative content in leaf volatiles) is a key discovery. o-Xylene is an aromatic hydrocarbon that might be involved in host location by females. Given that gravid females must ultimately select oviposition sites, this strong attraction raises the interesting hypothesis that o-xylene could function as an oviposition cue. However, because our field experiment only measured adult trap catch and did not observe subsequent oviposition behavior, this interpretation remains speculative and requires validation through targeted behavioral assays (e.g., oviposition choice tests) in future work. Female *N. hequensis* adults use contact chemoreception and olfaction to select oviposition sites; the presence of specific host volatiles indicates leaf quality and low competition. In contrast, male *N. hequensis* adults were most attracted to (*E*)-2-Hexenal, a ubiquitous green leaf volatile released from damaged or healthy leaves. This compound may indicate the presence of a host plant with active feeding (by conspecifics or other herbivores) and could serve as a rendezvous signal for mating. Such sex-specific divergence in odorant preference has been documented in other tenthredinids, e.g., *Hoplocampa testudinea* (apple sawfly) where female adults respond to floral volatiles and male adults to leaf volatiles. The practical implication is that we can develop gender-specific lures: *o*-Xylene for *N. hequensis* female adult biased trapping (which could reduce future population growth by removing reproductive *N. hequensis* female adults), and (*E*)-2-Hexenal for *N. hequensis* male adult monitoring (which is useful for early detection and phenology prediction). The *N. hequensis* male adult biased trap catch overall (4.5:1 ratio) reflects the natural sex ratio and suggests no strong behavioral bias beyond what is expected from population demography.

### 4.3. Trap Type Comparison

The large boat-shaped trap outperformed the triangular trap by a factor of 4.8. This difference is likely due to two design features: (1) larger sticky board area (800 cm^2^ vs. 450 cm^2^), (2) four open entrances allowing approach from all directions vs. only two ends. The poor performance of triangular traps suggests that *N. hequensis* adults are not strongly attracted to the physical shape or color of triangular traps, and rely more heavily on olfactory cues. However, because triangular traps are cheaper (US$0.7 vs. US$1.5) and easier to install and maintain, they could be deployed in large numbers for low-density monitoring if the target is only males and the user accepts low catch rates.

### 4.4. Effect of Hanging Height

The experimental results showed that the combination of a 1.5 m suspension height, bucket-type traps, eraser-type slow-release lures, and slow-release bag lures did not significantly attract adult *N. hequensis* in the field. The total number of captured adults was <30, and none of the individual experimental groups showed any significant differences. Although these results deviated from the experimental expectations, they provide valuable references for future experimental designs and a deeper understanding of the biological characteristics of adult *N. hequensis*. When the traps were suspended at a height of 1.5 m, the total number of adult *N. hequensis* captured was <70. This is partly due to the weak flying ability of adult *N. hequensis* and because they tend to be active at lower heights owing to food sources, habitat preferences, or strategies to avoid predators. In some field-trapping experiments, the number of target insects captured by the traps gradually decreased with an increasing suspension height, and in some cases, the traps could not effectively capture the target insects. For example, in studies targeting *Chilo suppressalis* (Walker), the number of captured adults gradually decreased with an increasing trap suspension height [12]. Lure effectiveness is influenced by the suspension height. For example, in field-trapping experiments using lures containing plant volatiles for *Monochamus alternatus*, the attractants released by lures at higher heights could not be sufficiently dispersed or did not reach areas where the pests were active. Consequently, the pests could not detect the presence of attractants and were thus not attracted to the traps [11]. In addition, higher lure positions are more susceptible to wind effects, which can alter the concentration and diffusion range of attractants, making it difficult for pests to be lured into the traps [13].

### 4.5. Effects of Environmental Factors on the Volatile Components of Host Plants

The composition of volatiles from the same plant species can vary in terms of composition and content depending on the geographical environment, season, growth stage, and organ location. Bai’s research showed that seasonal changes and daily variations can affect the release of volatile substances in subtropical bamboo forests [14]; Wu’s analyses revealed significant differences in the types and content of volatile substances in the tender shoots of *Citrus reticulata* across the different months [15]; and the composition of volatiles in *Zizyphus jujuba* differs significantly during the young leaf, flowering, and young fruit stages. The variety of volatiles during the young fruit stage is much greater than that during the young leaf stage, and each developmental stage harbors its own unique volatiles [16]. Considering the above factors, volatile samples were collected in the present study during the adult occurrence period of *N. hequensis*. The collection period was from 10:00 to 15:00, which is the peak activity period for *N. hequensis* adults. The collected samples were mature, healthy tender leaves sourced from an area in which they exhibited long-term occurrence.

### 4.6. Influence of Environmental Factors on the Effect of Field Trapping

Environmental factors have a significant impact on the field-trapping efficiency of insects. Temperature, humidity, light, wind speed, vegetation coverage, and hanging height are important influencing factors [17]. Suitable temperature and humidity can promote the activity and foraging behavior of insects, thereby enhancing trapping efficiency [18]. Light intensity and photoperiod significantly affect the activity and orientation of certain insects, and appropriate lighting conditions can significantly increase the capture rate of traps [19]. High wind speeds may hinder the flight of insects and affect trapping efficiency, whereas under lower wind speed conditions, the capture rate of traps is higher [20]. Vegetation cover and plant species have important effects on the habitat and environmental activity of insects. Suitable vegetation cover can enhance the capture rate of traps [10]. Therefore, in practical applications, comprehensively considering the impact of various environmental factors and choosing appropriate trapping strategies and methods to improve trapping efficiency are crucial.

### 4.7. Comparison with Other Sawfly Attractant Studies

Only a handful of studies have developed plant volatile attractants for sawflies. For the pine sawfly *Neodiprion sertifer* (Diprionidae), (*E*)-β-farnesene and other terpenes have been identified as attractants [21]. Within the family Tenthredinidae, the apple sawfly *Hoplocampa testudinea* has been shown to respond to pear ester (ethyl (E,Z)-2,4-decadienoate), which is primarily considered a feeding or oviposition stimulant derived from its rosaceous host plants [22]. In contrast to these prior reports, our study reveals two distinctive features for *N. hequensis*: (1) the attractants are derived from Salicaceae hosts, representing a different chemical landscape (green leaf volatiles and aromatic hydrocarbons rather than esters or terpenoids); and (2) we observed a clear sex-specific dichotomy, which has not been previously documented in tenthredinid attractant studies. This divergence suggests that the chemical ecology of host location varies substantially within the family, likely shaped by the specific host-plant chemistry and the pest’s life history. However, given the limited number of species studied, our findings underscore the need for more comparative research on sawfly–host volatile interactions.

### 4.8. Study Limitations and Future Directions

Several limitations must be acknowledged. First, our field experiments were conducted at a single site (Lhasa, Tibet, 3650 m altitude) during a single field season (August–September 2024). This high-altitude site has a specific microclimate (mean temperature 14.2 °C, relative humidity 63%) that may not be representative of other regions where *N. hequensis* occurs, such as lower-altitude areas in Liaoning or Inner Mongolia with warmer temperatures and different humidity regimes. While we designed the experiment with three replicate blocks and weekly trap rotations to minimize spatial and temporal variability within the site, our results cannot be generalized to other environments without further validation. Multi-site, multi-year studies are essential to confirm the robustness of our optimal trap configurations across the species’ geographic range. Second, we did not measure real-time volatile release rates under field temperature and wind conditions; as the reviewer rightly pointed out, environmental factors such as temperature, humidity, and wind speed can substantially affect the evaporation and dispersion rates of semiochemicals [11,13]. Higher temperatures may accelerate release rates, potentially shifting the effective concentration away from the 10 μg/μL optimum we identified, while stronger winds may dilute the volatile plume or alter its spatial structure, reducing trap attraction. Future studies should therefore monitor local weather conditions and consider adjusting lure concentration, release rate, or replacement frequency based on ambient temperature and wind conditions. Third, we did not test blend effects (mixtures of compounds). In nature, insects respond to complex blends, and a synthetic blend might be even more attractive than single compounds. Fourth, we did not assess the longevity of lures under field conditions beyond four weeks; longer-duration studies are needed for operational use. Fifth, the bucket traps and eraser/bag lures performed poorly in preliminary tests, but we did not systematically optimize them; there may be alternative designs or release materials that work better; Sixth, the negative control lures used in this study contained natural honey (from *Lepidotrigona arciferal*) as a carrier for the volatile compounds. While honey is commonly used in insect attractant studies as a feeding stimulant or slow-release matrix, it may itself exert a weak attractive effect on some insects. However, the capture numbers in our blank control treatments were consistently low across all experimental configurations, suggesting that any such effect was minimal and did not materially influence the comparative results among the tested volatile compounds. Nevertheless, we acknowledge this as a minor caveat for the sake of transparency; Seventh, purity verification of commercial volatiles. All 15 volatile compounds used in this study were purchased from commercial suppliers with declared purities ≥ 97%. However, we did not independently verify the purity of these compounds by GC-FID or GC-MS prior to field deployment. We acknowledge this as a methodological limitation, as even minor impurities (<3%) can, in some cases, produce confounding behavioral effects. For example, [23] demonstrated that a trace impurity in a commercial attractant formulation (*Z*)-5-decenyl acetate attracted a non-target moth species instead of the intended target, highlighting the importance of rigorous purity verification in behavioral studies. In our case, two lines of evidence suggest that the observed sex-specific attraction is primarily attributable to the target compounds rather than to shared impurities: (1) the two key attractants (*E*)-2-hexenal and o-xylene originate from different suppliers and different chemical classes, making a common contaminant unlikely to explain the divergent male vs. female responses; and (2) the concentration-dependent response pattern (peak at 10 μg/μL) is consistent with the known olfactory physiology of the species, as supported by our previous EAG recordings [10]. Nevertheless, we strongly recommend that future studies employing these or similar volatiles perform independent purity verification by GC-FID or GC-MS, and consider analytical assessment of lure stability and degradation products over time under field conditions.

Future research should: (1) test the optimal combinations in other regions (e.g., lower altitudes, different climates); (2) systematically evaluate volatile blends, with particular emphasis on the (E)-2-hexenal and o-xylene combination. Because our results revealed clear sex-specific preferences, we caution against assuming that a simple 1:1 mixture will necessarily enhance attraction. Instead, future blend experiments should test multiple ratios (e.g., 9:1, 1:1, 1:9) to identify whether synergistic effects exist or whether one compound interferes with the other’s attractiveness. Additionally, incorporating minor components from the 15-compound profile (e.g., 2-hydroxybenzaldehyde or ethylbenzene) into the blend may more closely mimic the natural host odor and potentially improve overall trap catch. Such blend optimization is the logical next step now that the most effective single compounds and their optimal concentrations have been established; (3) develop a controlled-release formulation with extended field life (e.g., 8–12 weeks); (4) assess non-target effects on beneficial insects; and (5) integrate attractant-based monitoring into decision-supported Integrated Pest Management (IPM) programs.

## 5. Conclusions

This study successfully developed and field-optimized plant volatile-based attractants for *N. hequensis* adults. The key findings and recommendations are: (1) Optimal configuration for general monitoring (both sexes): large boat-shaped traps, hanging height 1.0 m, spacing 10 m (one per 100 m^2^), slow-release bottle lures, with a choice of attractant depending on target sex; (2) For male-focused monitoring (early detection, phenology): use 10 μg/μL (*E*)-2-Hexenal. Expected capture: 265 males per trap over 4 weeks; (3) For female-focused monitoring (population suppression): use 10 μg/μL *o*-Xylene. Expected capture: 62 females per trap over 4 weeks; (4) Low-cost alternative for males only: triangular trap with 0.1 μg/μL ethylbenzene, but captures will be 30 males per 4 weeks, only 11% of the boat trap efficiency; (5) Non-linear concentration response: 10 μg/μL is optimal; higher concentrations are less effective.

These results provide the first plant-volatile attractant specifically developed for the genus *Nematus* and for a sawfly pest on Salicaceae hosts. The strong sex-specific responses also offer new insights into the olfactory ecology of tenthredinids. Practically, this study provides a ready-to-use, environmentally friendly monitoring tool for *N. hequensis* in the Tibetan region and potentially beyond. The sex-specific responses also shed light on the chemical ecology of host location in sawflies. With further validation across regions and development of longer-lasting lures, this technology could potentially be integrated into IPM programs for willow pests in the Tibetan region and beyond.

## Figures and Tables

**Table 1:** Volatile compounds derived from *S. alba* leaves.

Serial Number	Compound	Supplier
1	Dimethyl sulfide	Chengdu Alfa Biotechnology Co., Ltd. (Chengdu, China)
2	(*E*)-2-Hexenal	Chengdu Alfa Biotechnology Co., Ltd. (Chengdu, China)
3	Ethylbenzene	Chengdu Alfa Biotechnology Co., Ltd. (Chengdu, China)
4	*p*-Xylene	Chengdu Alfa Biotechnology Co., Ltd. (Chengdu, China)
5	*o*-Xylene	Chengdu Alfa Biotechnology Co., Ltd. (Chengdu, China)
6	Styrene	Chengdu Alfa Biotechnology Co., Ltd. (Chengdu, China)
7	Benzaldehyde	Chengdu Alfa Biotechnology Co., Ltd. (Chengdu, China)
8	Furan, 2-pentyl-	Chengdu MingRui Biotechnology Co., Ltd. (Chengdu, China)
9	(*Z*)-3-Hexen-1-ol acetate	Chengdu MingRui Biotechnology Co., Ltd. (Chengdu, China)
10	Benzyl alcohol	Chengdu Alfa Biotechnology Co., Ltd. (Chengdu, China)
11	2-Hydroxybenzaldehyde	Chengdu MingRui Biotechnology Co., Ltd. (Chengdu, China)
12	2-Ethyl-1,6-dioxaspiro[4.4]nonane	Dr. Ehrenstorfer (Augsburg, Germany)
13	Butanoic acid, (*Z*)-3-hexenyl ester	Chengdu MingRui Biotechnology Co., Ltd. (Chengdu, China)
14	Cis-3-hexenyl isovalerate	Chengdu MingRui Biotechnology Co., Ltd. (Chengdu, China)
15	Pentadecanal-	Chengdu Alfa Biotechnology Co., Ltd. (Chengdu, China)

Note: All compounds were purchased from commercial suppliers with declared purities ≥97%. RI and relative content data were previously reported in our companion study [10].

**Table 2:** Three trap types were used.

Model	Explanation
Large boat-shaped trap (model YL-LWT-1)	The green base is equipped with a 40 × 20 cm rectangular viscous plate with four open entrances (two 20 × 10 cm and two 40 × 10 cm). The lure is placed in the center.
Triangular trap (model YL-DT-2)	White triangular prism (30 cm length, 15 cm height), two open ends, the interior contains a replaceable sticky board (30 cm × 15 cm). The lure is placed in the center.
Bucket trap (model BT-L-P2.0)	Plastic bucket (15 cm diameter, 15 cm depth) with a funnel top. The bucket is partially filled with water (0.5 L) plus a drop of dish soap to reduce surface tension. The lure is hung 5 cm below the funnel. Captured insects fall into the water and drown.
Attractant cores	Eraser-type slow-release (model Y, 1 cm diameter × 1.5 cm height), bag-type (model YL, a porous polyethylene sachet 4 × 3 cm), and bottle-type (model YL-2ml, 2 mL capacity, capacity polyethylene bottle with a 0.5 mm diameter release hole). All traps and cores were from Zhongjie Sifang Co., Beijing.

## Data Availability

All data generated or analyzed in this study are included in this published article.

## References

[1] Xiao G. (1990). Four new species of Chinese sawfly (Hymenoptera, Platycladidae: Tenthredinidae, Tenthredinidae). For. Sci. Res..

[2] Lei X., Ci R., Zhao Y., Wang W., Zhang H., Liu H., Yao X., Pang B. (2021). Occurrence and control measures of *Nematus hequensis* Xiao in Lhasa. J. Anhui Agric. Sci..

[3] Lei X., Pang B., Zhuo G., Ci R., Li Y., Yao X. (2019). Occurrence and control strategies of *Nematus hequensis* Xiao in Lhasa. Plant Prot..

[4] Du X. (2022). Occurrence and control methods of *Nematus hequensis* Xiao in Xixia County, Henan Province. Spec. Econ. Anim. Plants.

[5] Wang H., Feng S., Liu X., Yu G. (2015). Morphological characteristics, habits and control strategies of *Nematus hequensis* Xiao. Environ. Entomol..

[6] Feng R., Gao Z. (2012). *Nematus hequensis* Xiao Integrated Control Technology Trial. Agric. Technol. Inf..

[7] Zhu B., Liang P. (2024). Occurrence and management of pest resistance to insecticides. Mod. Agrochem..

[8] Zhang Z., Jin Q., Wang F., Wang X., Wang X. (2024). Research progress on the toxic effects of chemical pesticides on non-target organisms in rice-fish integrated farming. J. Hebei Norm. Univ..

[9] Li X., Zhang X.-G., Xiao C., Gao Y.-L., Dong W.-X. (2019). Behavioral responses of potato tuber moth (*Phthorimaea operculella*) to tobacco plant volatiles. J. Integr. Agric..

[10] Song Z., Chen Y., Dong X., Sun Z., Guo X., Yu K., Tang X., Zang J. (2025). Electroantennogram and Behavioral Responses of *Nematus hequensis* Xiao Adult to Volatiles of *Salix alba* L. leaves. Arch. Insect Biochem. Physiol..

[11] Hu Q., Jin J., Du Y., Fan J. (2018). Changes in attractant components in *Monochamus alternatus* lure cores under field conditions and their effects on trapping efficiency. Acta Entomol. Sin..

[12] Zhou S., Li L., Lu X., Wang Q., Luan L. (2020). Effect of trap type and hanging height on the attraction efficiency of *Chilo suppressalis (Walker)*. J. Northeast. Agric. Sci..

[13] Wang Y., Tang L., Yue C., Zhang J., Zhang X., Alimu, Liu A., Chao D. (2011). Effect of different hanging heights and field wind direction of sex pheromone on the trapping efficiency of *Oriental Fruit Moth*. Xinjiang Agric. Sci..

[14] Bai J., Guenther A., Turnipseed A., Duhl T., Yu S., Wang B. (2016). Seasonal variations in whole-ecosystem BVOC emissions from a subtropical bamboo plantation in China. Atmos. Environ..

[15] Wu L.H. (2019). The Olfactory Responses of *Diaphorina citri* to the Volatile Compounds of *Nanfeng, Mandarin*. Master’s Thesis.

[16] Han Y., Li X.G., Yang L.J., Fan Y.L., Zhang X.W. (2010). Volatiles of Chinese jujube during different developmental phases. J. Northwest For. Univ..

[17] Su J., Cai Z., Qiao F., Miao L., Yin S., Zheng P. (2020). Comparative study on the attraction effect of plant-derived volatiles on natural enemy insects in cornfields. J. Appl. Entomol..

[18] Xu X., Li X., Wang H., Zhang L., Pu P., Wang X. (2024). Construction of Prediction Model for Annual Induction Amount of *Sesamum Indicum* Moths in Main Rice Areas of Sichuan Province Based on Light and Sex Attraction Network. J. Sichuan Agric. Univ..

[19] Shang Y., Ni M., Jiang M. (2024). Thigmotaxis of *Callosobruchus maculatus* and *Acanthoscelides obtectus* (Say) to light sources of different wavelengths and illumination intensities. J. Environ. Entomol..

[20] Chen Y., Ma C. (2010). Research progress on the impact of climate warming on insects. Acta Ecol. Sin..

[21] Rahman-Soad A., Skuras L., Reinecke A., Varama M., Hilker M. (2024). Sawfly sex pheromones: Analysis of their impact on pine odor attractive to egg parasitoids. J. Chem. Ecol..

[22] Zsolt K., Béla P.M. (2022). Antennal responses of black plum sawfly (*Hoplocampa minuta*) to European plum (*Prunus domestica*) flower volatiles. Acta Phytopathol. Entomol. Hung..

[23] Nagy A., Szarukán I., Bohman B., Szanyi S., Kozák L., Szilágyi A., Imrei Z., Vuts J., Matula E., Varga Z. (2023). Catches of Euxoa tritici in pheromone traps for Anarsia lineatella are due to the presence of (Z)-5-decenyl acetate as an impurity. Entomol. Exp. Appl..

